# Revisiting in vivo staining with alizarin red S - a valuable approach to analyse zebrafish skeletal mineralization during development and regeneration

**DOI:** 10.1186/s12861-016-0102-4

**Published:** 2016-01-19

**Authors:** A. Bensimon-Brito, J. Cardeira, G. Dionísio, A. Huysseune, M. L. Cancela, P. E. Witten

**Affiliations:** Centre of Marine Sciences – CCMar, University of Algarve, Campus de Gambelas, Faro, Portugal; Evolutionary Developmental Biology, Biology Department, Ghent University, Ghent, Belgium; ProRegeM PhD Programme, Department of Biomedical Sciences and Medicine, University of Algarve, Campus de Gambelas, Faro, Portugal; Guia Marine Laboratory, Oceanography Centre, Faculty of Sciences of University of Lisbon, Cascais, Portugal; Department of Biomedical Sciences and Medicine, University of Algarve, Campus de Gambelas, Faro, Portugal; Current address: CEDOC - Faculdade de Ciências Médicas, Universidade Nova de Lisboa, Lisbon, Portugal

**Keywords:** Vertebral column, Caudal fin, Mineral apposition, Bone, Fluorescence imaging, Calcium, Hydroxyapatite, Alizarin red S

## Abstract

**Background:**

The correct evaluation of mineralization is fundamental for the study of skeletal development, maintenance, and regeneration. Current methods to visualize mineralized tissue in zebrafish rely on: 1) fixed specimens; 2) radiographic and μCT techniques, that are ultimately limited in resolution; or 3) vital stains with fluorochromes that are indistinguishable from the signal of green fluorescent protein (GFP)-labelled cells. Alizarin compounds, either in the form of alizarin red S (ARS) or alizarin complexone (ALC), have long been used to stain the mineralized skeleton in fixed specimens from all vertebrate groups. Recent works have used ARS vital staining in zebrafish and medaka, yet not based on consistent protocols. There is a fundamental concern on whether ARS vital staining, achieved by adding ARS to the water, can affect bone formation in juvenile and adult zebrafish, as ARS has been shown to inhibit skeletal growth and mineralization in mammals.

**Results:**

Here we present a protocol for vital staining of mineralized structures in zebrafish with a low ARS concentration that does not affect bone mineralization, even after repetitive ARS staining events, as confirmed by careful imaging under fluorescent light. Early and late stages of bone development are equally unaffected by this vital staining protocol. From all tested concentrations, 0.01 % ARS yielded correct detection of bone calcium deposits without inducing additional stress to fish.

**Conclusions:**

The proposed ARS vital staining protocol can be combined with GFP fluorescence associated with skeletal tissues and thus represents a powerful tool for in vivo monitoring of mineralized structures. We provide examples from wild type and transgenic GFP-expressing zebrafish, for endoskeletal development and dermal fin ray regeneration.

## Background

Skeletal mineralization relies on a tightly regulated connection between cell activity and extracellular environment. Researchers in skeletal biology analyse the cellular and molecular events underlying skeletal matrix formation and maintenance, and the mechanisms that promote and limit the mineralization of the matrix. Therefore, standardized methodologies and tools are a prerequisite to assess and quantify extracellular matrix mineralization in the context of bone and cartilage development, skeletal growth, remodelling and regeneration [1].

Teleost fish, such as zebrafish (*Danio rerio*), are recognized models to study skeletal development and regeneration [2]. The development of the skeleton can be observed at very early stages since embryonic/larval zebrafish remain translucent during the first important steps of skeletal development [3]. In addition, the complete genome sequence and its annotation are available, as well as a broad array of molecular and cellular tools. An increasing number of well characterized fish mutants has been derived from large scale mutagenesis screens ([4–6]; http://www.sanger.ac.uk/resources/zebrafish/zmp/), and many transgenic fish lines have been developed using fluorescent proteins (such as Green Fluorescent Protein - GFP) to report the expression of skeleton-related genes [7]. Recently, the development of reverse genetic approaches, such as TALE nucleases and Crispr/Cas9 systems, opened new horizons for targeted mutagenesis in zebrafish [8]. Overall, these advantages make zebrafish a valuable vertebrate model system, widely used in fundamental and applied research (reviewed by [2, 9, 10]).

The study of mineralized structures in teleost fish is traditionally based on the analysis of fixed samples [11–19]. For live imaging, bone development can be tracked with radiographs in large specimens [20], but for small sized species, such as zebrafish, the use of radiographic and μCT approaches to visualize the skeleton is restricted due to resolution constraints [1, 21, 22]. Thus, there is a need for reliable and non-toxic in vivo imaging techniques to allow continuous monitoring of skeletal development in living individual zebrafish.

Fluorescent calcium dyes (e.g., calcein, tetracycline, xylenol orange and alizarin red) can label calcium-containing tissues and be used to follow skeletal mineralization in vivo. Sclerochronology, in the frame of fish stock assessment, is a common application for calcium dyes [23–27]. For zebrafish, only the use of calcein has been optimized for in vivo staining [28] but most transgenic zebrafish lines use GFP as a reporter [7], which emits fluorescence within the same spectrum as calcein. In addition, the fluorescence spectrum of calcein is similar to that obtained with fish tissue autofluorescence [29]. Thus, alternatives to calcein for zebrafish skeletal staining are desirable.

Alizarin (1,2-dihydroxyanthraquinone), which emits a red signal under fluorescent green light, has been used for in vivo labelling for many decades [30]. Vital staining of fish bone is accomplished with two Alizarin variants, Alizarin red S (ARS) and alizarin complexone (ALC). In a study on Japanese flounder *Paralichthys olivaceus* [31] similar concentrations of ALC and ARS (300 mg/l ALC and 400 mg/l ARS) were shown to provide equally strong staining by fish immersion in the staining solution. Several studies performed on zebrafish and medaka also show the applicability of in vivo alizarin skeletal staining (Table 1). Yet, published protocols suffer from two shortcomings. First, a consistent protocol concerning alizarin concentration, time of immersion and washing steps has not been established. Second, possible negative effects of alizarin on bone growth and mineralization have not been assessed. Since alizarin has been described to inhibit growth and mineralization in vivo in rats, rabbits and guinea-pigs [11], a careful validation of alizarin live staining protocols is required.

The results of this study show that ARS, used according to the protocol defined in the present work, is a reliable tool for in vivo staining and detailed analysis of mineralized skeletal structures in developing and in adult zebrafish. A detailed quantitative analysis of growth and mineral apposition rates revealed that a low concentration of ARS, combined with short immersion intervals, has no negative effect on bone development. A standardized staining protocol is suggested and its applicability is demonstrated on the developing skeleton of zebrafish and on regenerating caudal fin rays (lepidotrichia) in adult zebrafish.

**Table 1:** Overview of studies using in vivo staining with alizarin compounds (ALC and ARS) by immersion for in vivo skeletal analysis or paraformaldehyde fixed teleost specimens. Species names, dye concentrations, duration of immersion, wash steps, and literature references are indicated

	Alizarin compound	Concentration	Time of immersion & washing	Species	Reference
in vivo	ARS	0.003 %	2–3 h / rinsing	*Danio rerio*	DeLaurier et al. 2010 [53]
	ALC	0.005 %	O/N / rinsing	*Oryzias latipes*	Renn et al. 2013 [54]
	ALC	0.005 %	n.d. / rinsing	*Oryzias latipes*	Inohaya et al. 2007 [55]
	ARS	0.005 % + HEPES	Larvae 1–2 h; juvenile ON / rinsing	*Danio rerio*	Kimmel et al. 2010 [56]
	ALC	0.010 %	2 h / rinsing	*Oryzias latipes*	Willems et al. 2012 [57]
	ALC	0.010 %	2 h-4 h / 2 h-ON	*Oryzias latipes*	To et al. 2012 [58]
	ARS	0.020 %	10 min / rinsing	*Danio rerio*	Tu and Johnson 2011 [38]
	ALC	0.025 %	n.d.	*Danio rerio & Oryzias latipes*	Chatani et al. 2011 [59]
	ARS	0.025 %	24 h / rinsed	*Poecilia reticulata*	Bashey 2004 [29]
	ARS	0.040 %	n.d. / rinsing 10 min	*Danio rerio*	Recidoro et al. 2014 [60]
	ARS	0.050 %	5 min / rinsing	*Danio rerio*	Huitema et al. 2012 [61]
	n.d.	0.050 %	n.d.	*Danio rerio*	Fleming et al. 2004 [62]
	n.d.	0.300 %	n.d.	*Danio rerio*	Eames et al. 2010 [63]
	n.d.	n.d.	n.d.	*Danio rerio*	Yan et al. 2005 [64]
Post mortem	ALC	0.0025 %*	6 h / n.d.	*Theragra chalcogramma*	Dougherty 2008 [65]
	ALC	0.003 %	24 h / n.d.	*Acanthopagrus butcheri*	Partridge et al. 2009 [66]
	ALC	0.003 %*	12 h / n.d.	*Argyrosous japonicus*	Taylor et al. 2005 [32]
	ALC	0.006 %*	6–24 h / n.d.	*Scophthalmus maximus*	Iglesias et al. 1997 [67]
	ARS	0.010 %	12 h / n.d.	*Clupea harengus*	Bang et al. 2007 [68]
	ALC	0.010 %	23 h / n.d.	*Esox lucius*	Skov et al. 2001 [69]
	ALC	0.012 %	24 h / n.d.	*Scophthalmus maximus*	Lagardère et al. 2000 [33]
	ARS	0.040 %*	24 h / n.d.	*Scophthalmus maximus*	
	ARS	0.015 %*	3 h / n.d.	*Salmo trutta*	Baer and Rosch 2008 [70]
	ALC	0.025 %	15 min / n.d	*Oryzias latipes*	Nemoto et al. 2007 [71]
	ALC	0.030 %*	24 h / 4 h	*Paralichthys olivaceus*	Liu et al. 2009 [31]
	ARS	0.040 %*	24 h / 4 h	*Paralichthys olivaceus*	

An asterisk denotes the concentrations considered the most effective from a range of concentrations tested in the referred studies. *n.d*. not defined

## Results and discussion

### Alizarin red S in vivo staining - exploring optimal concentrations

**Fig. 1:**
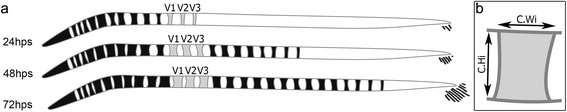
Quantification of mineral apposition in developing zebrafish larvae. Schematic representation of the quantification of the mineral apposition rates in vertebral centra following ARS or calcein staining. **a** Mineral apposition was determined (at 24, 48 and 72 h post-staining - hps) by monitoring the mineralized surface areas (SA’s) of the three least mineralized vertebral centra (*grey*) in the beginning of the experiment. **b** Centra SA’s were calculated based on width (C.Wi) and height (C.Hi), as indicated

Proper staining of skeletal elements in fish by immersion in fluorochrome solutions demands a compromise between concentration, immersion period, survival and rearing conditions [32]. Currently, most protocols used for vital staining of bone rely on Alizarin compounds. However, the published protocols vary concerning dye concentration and time of immersion (Table 1). Despite the potential that ALC may have, this study focused on testing a single compound, ARS, to simplify the analysis. We also aimed at developing a protocol with short immersion periods, in contrast to existing protocols, in which immersion takes up to 24 h [29, 31, 33]. Here, a daily, single immersion period of 15 min is proposed both for larvae (Fig. 1) and adult zebrafish. Calcein, another standard reagent for in vivo skeletal staining of zebrafish [28], was used as a control staining, following an established calcein staining protocol [28]. It should, however, be noted that ARS concentrations used here were much lower (0.005 to 0.05 %) than those used for calcein (0.2 %).

**Fig. 2:**
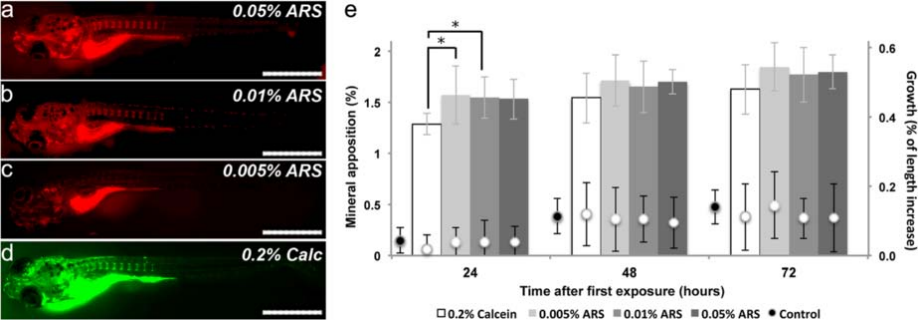
Determination of the proper ARS concentration for vital staining. Imaging of 6 dpf stained larvae with the same settings showed that (**a**) 0.05 % ARS 15 min immersion yielded stronger staining than (**b**) 0.01 % ARS, but the later provided the best signal to noise ratio, with minimum stress levels. **c** 0.005 % ARS was considered the lowest concentration providing signal detection, since most structures were weakly stained. **d** 0.2 % calcein staining was used as a reference staining. **e** Graphical representation of mineral apposition rates (columns) at 24, 48 and 72 h after first staining, when exposed to 0.005, 0.01 and 0.05 % ARS and 0.2 % calcein. Bars represent standard deviation. Means were statistically different (*p < 0.05), by multiple comparison of means using one-way ANOVA and Tukey’s post test, between larvae stained with calcein and those stained with 0.005 % (0.29 % less apposition rate with calcein, 82 % of the 0.005 % ARS value) and 0.01 % (0.26 % less mineral apposition rate with calcein, 83 % of the 0.01 % ARS value) ARS at 24 hps. On the second axis of the graph, growth (inferred by increase in TL) is indicated: control conditions (black dots; *n* = 17); following staining with 0.005, 0.01 and 0.05 % ARS, and 0.2 % calcein (*white dots*; *n* = 17). Scale bars = 1 mm

Using the same imaging settings (Fig. 2), 0.05 % ARS yielded a strong mineral staining (Fig. 2a), but at 5× lower concentrations, 0.01 % ARS, lighter but still adequate detection of all mineralized structures were observed (Fig. 2b). In contrast, 0.005 % ARS yielded only a sparse and incomplete detection of mineralized structures, with evident false negative staining (Fig. 2c). Accordingly, a 15 min exposure to 0.005 % ARS is inappropriate for the labelling and subsequent correct detection of mineralized structures. The highest tested ARS concentration (0.05 %), although providing strong staining, caused significant stress to fish, particularly in adults, noted by direct observation of increased operculum movements [34].

We also observed that calcein (Fig. 2d), under the established concentration [28], displayed a higher background staining when observed with epifluorescence compared to all ARS concentrations. To eliminate the background staining, calcein stained specimens required additional, time consuming, rinsing steps.

Next, we tested if different concentrations of ARS and calcein affected mineral apposition and animal growth (Fig. 1). We did not observe significant differences in growth rate either among ARS treated larvae, or when comparing ARS-treated, calcein-treated and control groups (Fig. 2e). This shows that none of the staining protocols has a detectable effect on growth. For mineral apposition rates, differences between fish stained with calcein and ARS were registered at 24 h after first exposure. Developing vertebral centra exposed to 0.2 % calcein showed approximately 82 % of the mineral apposition rate registered with ARS, corresponding to a decrease of 0.29 % detected mineral when compared with 0.005 % ARS (*p* < 0.05), 0.26 % when compared with 0.01 % ARS (*p* < 0.05), and 0.24 % when compared with 0.05 % ARS. As there was no significant effect on growth rate, only the detected mineralization was affected by calcein.

At 48 and 72 h after first exposure, no significant differences were observed on mineral apposition rates between the three ARS protocols, showing that fish exposed to these concentrations of ARS did not suffer from inhibition of growth or mineral apposition rates, when compared with control and calcein stained fish.

This study shows that mineralization is not significantly affected when fish are treated daily for 15 min with low concentrations of ARS (i.e., ranging from 0.005 to 0.05 %), even if the treatment is repeated over several consecutive days. We propose the use of 0.01 % ARS as vital stain for bone during early and late skeletal development. This low ARS concentration provides clear staining of bone with no apparent induction of stress. The data on calcein staining suggest a mineralization inhibition at 24 h after first exposure, possibly due to the high concentration of the staining solution when compared with the tested ARS solutions. Furthermore, the green fluorescent signal from calcein and GFP reporter lines, which emit at a similar wavelength, are indistinguishable, reinforcing the value of ARS staining as an alternative to calcein.

### ARS staining of regenerating caudal fin lepidotrichia

**Fig. 3:**
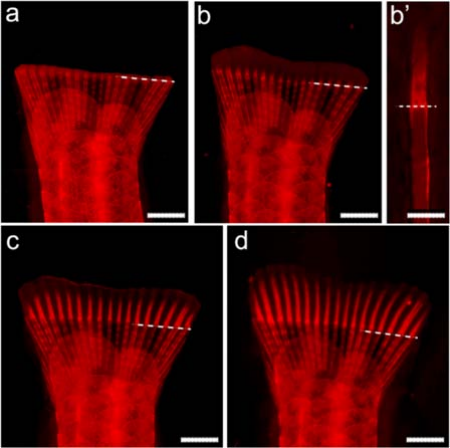
Sequence of lepidotrichia regeneration events in the zebrafish caudal fin. Caudal fin of fish stained with 0.01 % ARS at **a** 24, **b** 48, **c** 72 and **d** 96 hpa. **b’** Detail of a fin ray at 48 hpa, already displaying *de novo* mineralized tissue. Amputation axis is indicated (*dashed line*). Scale bar (**a-d**) = 2 mm; (**b’**) = 0.2 mm

One of the main topics of current caudal fin regeneration research is the differentiation of scleroblasts, the cells responsible for the formation of the mineralized matrix of the lepidotrichia [35–38]. In one published study, ARS was used together with GFP reporter lines, at a concentration of 0.02 % and an incubation time of 10 min [38]. In our study we also tested 0.01 % ARS staining, which was found to provide sufficient detection of mineral in the regenerating fin rays as early as 48 h post-amputation (hpa; Fig. 3).

### ARS detection sensitivity in fixed specimens

**Fig. 4:**
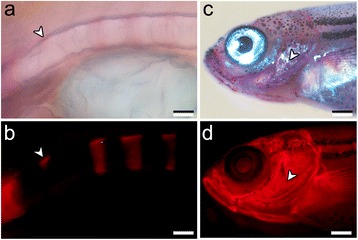
ARS staining of fixed zebrafish samples. Panels **a**-**b** show a vertebral column of a 10 dpf larva stained with 0.01 % ARS in 70 % ethanol. **a** Bright field observation provides less detail of the early mineralization deposits than **b** fluorescence observation (*arrowheads*). Panels **c**-**d** show cranial structures of a juvenile (30 dpf, 8 mm TL) stained with 0.01 % ARS and observed under **c** bright field and **d** fluorescent light, evidencing the higher power of detection of, e.g., the operculum (*arrowheads*) under fluorescent conditions. Scale bars (**a**, **b**) = 0.04 mm; (**c**, **d**) = 0.2 mm

**Fig. 5:**
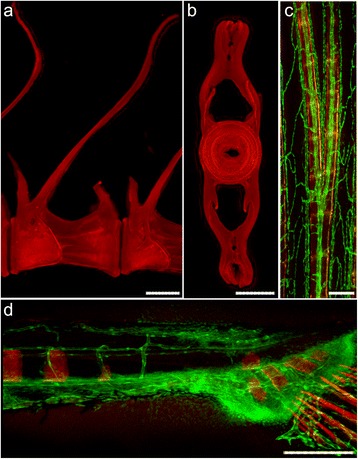
ARS fluorescence sensitivity single or in combination with expression of green fluorescent reporters. Macerated abdominal vertebrae of adult fish in **a** sagittal view and **b** transverse view show distinct mineralization fronts, indicative of vertebral growth. **c** Caudal fin ray of an adult Tg(*fli1:eGFP*) fish stained with 0.01 % ARS and **d** caudal vertebrae formation in a Tg(*fli1:eGFP*) zebrafish larva. There is a clear distinction between structures stained with ARS and structures that express GFP. Scale bars (**a**, **b**) = 0.1 mm; (**c**, **d**) = 0.2 mm

Alizarin compound staining procedures are commonly analysed using transmitted light source within the visible spectrum. In addition, it is also possible to take advantage of the fluorescent property of ARS. In published protocols, vitally stained specimens are fixed and subsequently analysed (Table 1). A more recent study [39] analyses ARS signals with fluorescent light in zebrafish that are stained after fixation. The authors describe as advantages of alizarin fluorescence the detailed assessment of mineralized structures without the need for advanced maceration of soft tissues. However, the staining solution used in this study was 0.1 % ARS dissolved in a 1.0 % KOH solution, which inevitably macerates the tissues due to its extreme high pH. We therefore tested on fixed samples the use of 0.01 % ARS concentration in a combination of short (15 min) immersion period with a staining solution without KOH addition. For this experiment, ARS was dissolved in an alcoholic solution [40]. This procedure offers a major advantage, particularly when combined with immunofluorescence (data not shown), since ARS staining without maceration of soft tissuesprovides a reliable co-localization of specific proteins and mineralized matrix. In addition, ARS staining tracked under fluorescence allows a detailed identification of early mineralization events, with a good signal/noise ratio. Fish larvae stained with this protocol can even be subjected to further histological analysis of tissues and cells [1, 41]. We can also observe the formation of vertebral centra anlagen within the notochord sheath that are difficult to observe with visible light (Fig. 4a, b), as well as structures of the head skeleton in larger specimens (Fig. 4c, d). With visible light, these bones are neither visible nor distinguishable from the background. In addition, ARS detected under fluorescence, proved to be suitable for the detailed observation of skeletal microstructures, such as vertebral body endplate growth rings (Fig. 5a, b). The repeated analysis of zebrafish early vertebral body mineralization in early developmental stages [17, 42, 43] revealed that, the exposure of specimens to KOH solutions, causes maceration of the mineralized matrices, such as the notochord sheath. Since the anlagen of the teleost vertebral centra form in the notochord sheath [44, 45], removal of the matrix through maceration is likely the cause for false negative ARS staining. So far, false negative ARS staining has only been recognized in connection to acid pre-treatment of fish larvae, in the course of double staining for cartilage and bone [1, 12, 39, 46, 47].

### Combination of ARS in vivo staining with GFP reporter lines, and a tool to reveal skeletal malformations

**Fig 6:**
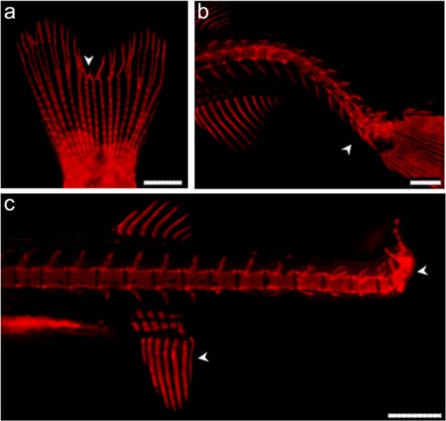
Detection of skeletal malformations in zebrafish. Deformed bony structures in **a** caudal fin rays and **b**-**c** different regions of the vertebral column. All regions display affected structures with different degrees of severity. *White arrowheads* show sites of malformation. Scale bars (**a**) = 2 mm; (**b**-**c**) = 0.4 mm

Here we tested the use of the proposed ARS staining protocol combined with a GFP reporter transgenic line. The direct observation of early mineralizing structures was shown to be possible in Tg(*fli1:egfp*) transgenic fish, which contain a reporter for the vascular system (Fig. 5c, d). The dual visualization of mineralization and GFP expression can be relevant for the documentation of bone pathologies. Indeed, skeletal malformations in fish are a subject of growing interest, related to malformations in farmed fish and to skeletal defects of model fish in the frame of biomedical research [6]. The example of malformed regenerated lepidotrichia shows that deformities can be observed in great detail using ARS staining (Fig. 6a). Further examples are the visualisation of lordosis (V shaped curvature) and kyphosis (Λ shaped curvature) in the vertebral column of juvenile zebrafish (Fig. 6b, c). The ARS in vivo staining permits the evaluation of live fish and the continuous tracking of malformations.

## Conclusions

Even though ARS has been used in the past as a fluorescent mineralization label [11, 39, 48], no consistent in vivo staining protocol has been proposed for small teleosts such as medaka or zebrafish. Here, such a protocol is proposed (Table 2). Our results show that, when applied in low concentrations (0.01 %) in combination with short-term immersion (15 min), ARS does not inhibit mineralization in developing zebrafish or in adult fish during fin ray regeneration. The quantification of mineralization allowed assessing the effect of repetitive staining on the progress of mineralization during development. It is shown here that the proposed ARS staining protocol can be safely used for repetitive staining procedures. It is also shown that ARS in vivo staining can be combined with detection of GFP reporter expression in transgenic lines and allows a detailed analysis of skeletal development and malformations. For staining of fixed specimens, we show that not only acid pre-treatment but also maceration with strong alkaline solutions can cause false negative staining of early mineralization. As an alternative to calcein staining, a standardized use of the proposed ARS staining protocol can provide detailed insights into skeletal development of small model organisms such as zebrafish and medaka.

**Table 2:** Bench protocol. Steps of the proposed ARS in vivo staining protocol

1. Prepare a 0.01 % ARS solution, using water from the system in which fish were previously maintained (system water or embryo medium)
1.1. A 5× concentrated solution (0.05 %) can be prepared with distilled water, then diluted in embryo medium or system water to 0.01 % working solution before use
1.2. Adjust pH to 7.4 with KOH solution
1.3. Keep solution in the dark when storing
2. Transfer fish to ARS solution
2.1. Adult specimens can be transferred with fish nets
2.2. Larval specimens can be transferred using Pasteur pipettes
3. Stain for 15 min with ARS solution
4. Rinse at least 3 times for 5 minutes in embryo medium or system water
4.1. Substitute staining solution with new embryo medium or system water, or transfer fish into new containers, as described in points 2.1. and 2.2.
5. Perform image analysis and photograph acquisition
5.1. Anaesthetize specimens with up to 0.6 mM MS222
5.2. Accommodate specimens for imaging (e.g., Petri dishes, glass-bottom dishes, excavated slides)
5.3. Use fluorescent microscope or stereomicroscope, depending on the desired magnification, coupled to the appropriate fluorescent filter
5.4. Image under green fluorescent light (510–550 nm)
6. Recover fish from anaesthesia, by transferring them to new embryo medium or system water

## Methods

### Ethics statement on animal experiments

Animal handling and experiments were accredited by the Portuguese Direcção Geral de Veterinária (DGV). All the experimental procedures involving animals followed the EU (Directive 2010/63/EU) and National (Decreto-Lei 113/2013) legislation for animal experimentation and welfare.

### Fish maintenance

Wild-type zebrafish (*Danio rerio*) ranging from 4.4 to 5.4 mm total length (TL) equalling 6 to 10 days post-fertilization (dpf), 30dpf juveniles, and three month old adult zebrafish, were maintained under standard conditions [3], with a photoperiod of 14 h light / 10 h dark. For staining experiments in developing fish, 6 to 10 dpf fish were incubated at 28° ± 1 °C in 24 well-plates (3 ml; 1 fish per well). During the experiments, larvae were fed daily with *Artemia* nauplii (*Artemia salina*) and rotifers (*Brachionus plicatilis*).

For the regeneration experiments, 3 months old adult fish were anaesthetised with 0.6 mM Tricaine (MS222; Sigma, St. Louis, MO) and caudal fin rays (lepidotrichia) were amputated one segment proximal to the first bifurcation. Fish were returned to their tanks and left to regenerate at 33° ± 1 °C, the accepted standard temperature for caudal fin regeneration studies [49, 50]. The fish were fed twice to satiation with commercial flakes (Benelux, Ooigem). The water was renewed daily, both for developing and adult specimens.

### ARS staining

For fixed samples, all specimens (at 10 dpf and 30 dpf and three month old fish) were euthanized with an overdose of MS 222 and subsequently fixed for 12 h in neutral buffered 4 % paraformaldehyde. All specimens were stained for 15 min with 0.01 % ARS (3,4-Dihydroxy-9,10-dioxo-2-anthracenesulfonic acid sodium salt, from Sigma-Aldrich, St. Louis, MO) dissolved in 70 % ethanol [40]. For a better visualization of the mineralized structures in adult fish, specimens were macerated with 3 % KOH for 12 h and subsequently dissected.

For vital staining, three ARS concentrations (0.005, 0.01 and 0.05 %) were prepared in embryo medium [3]. The pH was adjusted to 7.4 with KOH. No precipitated ARS occurred in any of the three concentrations.

For the study of bone development, the specimens were transferred with a minimum volume of embryo medium to a new 24-well plate [3] with 3 ml of staining solution or new embryo medium (control). The animals remained in the staining solution for 15 min. Staining was performed once a day from 6 to 10 dpf, in each of the three ARS solutions described above. 0.2 % calcein [28] was used as a reference dye for mineral staining. In this case, larvae were stained for 10 min, as previously described [28]. Following staining with ARS, larvae were rinsed in embryo medium 3 times for 5 min, while larvae stained with calcein had to be rinsed at least 3 times for 10 min with embryo medium. In all cases, we assured that no dye residues were externally visible after the last rinsing period. If so, additional rinsing was conducted.

Stress levels were assessed by observing variations in the opercular movement frequency, as previously described [34], upon fish immersion during the first minute of staining and for 1 min at end of the staining period, before rinsing. The remaining period (remaining staining periods, and washing steps) prior to skeletal tissue imaging, took place in a dark environment to avoid stress. However, our personal observations suggest that there is no apparent effect on staining efficiency or fish health if animals remain exposed to light.

For regeneration studies, 5 adult specimens (3 month old) were exposed for 15 min to 0.01 % ARS solution prepared in system water prior to amputation and every 24 h thereafter, until 96 h post amputation (hpa). Adult fish were rinsed 3 times after each staining event for 5 min also in system water.

After ARS and calcein staining, larvae and adult fish were kept for periods no longer than 30 min prior to imaging. All specimens were anaesthetised up to 0.6 mM Tricaine solution (MS222; Sigma, St. Louis, MO) prior to microscopy analysis. Imaging was performed under green (510–550 nm) and blue (450–480 nm) fluorescent light to image ARS and calcein staining, respectively, and under visible light for total length (TL) measurements. Images were captured using a Leica MZ6 stereo microscope (Leica Microsystems, Germany) equipped for epifluorescence together with a F-View II camera, and Cell^Fv2.7 software (Olympus Soft Imaging Solutions GmbH, Germany). Higher magnifications of skeletal structures were visualised using an Axio Imager Z2 microscope equipped with a digital AxioCam ICc3 camera (Zeiss, Germany).

Tg(*fli1:egfp*) transgenic fish [51] were used to validate the suitability of ARS vital staining applied to GFP labelled fish during the regeneration of the caudal fin rays and the development of caudal vertebrae.

ARS staining was also used to detect skeletal deformities. The analysed deformities were not induced, but developed under regular rearing conditions. All fish were photographed using the equipment and the procedures described above.

### Growth rate and mineral apposition in vertebral centra

In order to determine growth and mineral apposition rates, images of each specimen were taken using a Leica MZ6 stereo microscope (Leica Microsystems, Germany) for each time point, as described in the previous section.

The TL of individual fish was determined prior to immersion. TL was measured every day and the growth rate was calculated based on TL measured at each time point divided by TL at the beginning of the experiment.

Mineral apposition rates were assessed by tracing the area of three vertebral centra in each specimen in sagittal view (Fig. 1a; anterior-posterior axis). Due to individual variability and the increasing number of mineralized vertebral bodies in different developmental stages, it was not possible to track the development of the same vertebrae in all individuals. Therefore, the three least developed vertebrae in the abdominal region [43] were selected in each fish, which had equivalent areas of mineralization in all specimens at the start of the experiment. Nomenclature and histomorphometric methods were based on Parfitt’s standards [52]. On lateral microphotographs of vertebral bodies, the mineralized surface area (SA - Fig. 1b) of the centrum was determined by measuring centrum height (C.Hi) and width (C.Wi). Mineral apposition rates were determined by the quotient of the SA of the mineralized centrum at each time point and its initial SA (± standard deviation).

All measurements of growth and mineral apposition rates were performed using the software ImageJ 1.47d (Wayne Rasband, National Institutes of Health, USA). Digital measurements on highly enlarged photographs allowed a precision down to 0.1 μm.

All data were subjected to statistical analysis using GraphPad Prism software (version 4.0b). One-way ANOVA was used for the analysis of variance and Tukey’s post-test was used for multiple comparison of means.
